# Synergistic effects of 1-MCP and H₂S co-treatment on sugar and energy metabolisms in postharvest strawberry fruit

**DOI:** 10.3389/fnut.2025.1615783

**Published:** 2025-06-02

**Authors:** Xingyue Wang, Tian Qiu, JingJing Jiang, Kaili Shi, Zhikang Liu, YanYan Wang, Qingyuan Song, Chen Zhang, Tingyu Wu, Dan Chen, Xiaohui Yang, Fang Liu, Qing Gong, Wei Lan, Li Wang

**Affiliations:** ^1^Anhui Ecological Fermentation Engineering Research Center for Functional Fruit Beverage, School of Biology and Food Engineering, Fuyang Normal University, Fuyang, China; ^2^Key Laboratory of Agricultural Product Fine Processing and Resource Utilization, Ministry of Agriculture and Rural Affairs, Anhui Engineering Research Center for High-valued Utilization of Characteristic Agricultural Products, College of Food and Nutrition, Anhui Agricultural University, Hefei, China; ^3^College of Food Science and Engineering, Yangzhou University, Yangzhou, China

**Keywords:** 1-MCP, H_2_S, sucrose, energy status, strawberry

## Abstract

**Introduction:**

1-methylcyclopropene (1-MCP) and hydrogen sulfide (H_2_S) play important roles in the ripening and senescence of postharvest fruits and vegetables. However, little knowledge was available for the effect of the combined treatment of 1-MCP and H_2_S on the quality maintenance of postharvest strawberry fruit.

**Methods:**

The synergistic effects of 1.0 μL L^−1^ 1-MCP and 0.8 mmol L^−1^ sodium hydrosulfide (NaHS, H_2_S donor) combined treatment on the sugar and energy metabolisms of strawberry fruit during cold storage at 4 ± 0.5°C with a relative humidity of 80–85% for 15 d were studied.

**Results:**

The results showed that the combined treatment effectively suppressed the increase of decay rate, decay index, and weight loss rate while maintaining the firmness and visual quality of strawberry fruit compared to the 1-MCP or H_2_S treatment. Moreover, the combined treatment maintained higher sucrose content and lower contents of glucose and fructose by inhibiting the activities of acid invertase (AI) and neutral invertase (NI), while enhancing the activities of sucrose synthase (SS) and sucrose phosphate synthase (SPS). Meanwhile, strawberry fruit treated with the combination elevated ATP levels and energy charge by upregulating key enzymes in energy metabolism, including succinate dehydrogenase (SDH), cytochrome c oxidase (CCO), H^+^-adenosine triphosphatase (ATPase) and Ca^2+^-ATPase.

**Conclusion:**

These results indicated that 1-MCP and H₂S acted synergistically to regulate sugar catabolism and energy homeostasis, promoting elevated sucrose accumulation and mitochondrial energy production, thereby maintaining the integrity of cell structure and the quality of strawberry fruit.

## 1 Introduction

Strawberry fruit (*Fragaria × ananassa* Duch.) is a globally cherished commodity, prized for sensory attributes and nutritional richness, including high levels of vitamins, minerals, sugars, antioxidants and bioactive compounds (1). These components not only enhance its flavor profile but also confer antioxidant and anti-inflammatory benefits, contributing to its commercial significance (2). Nevertheless, postharvest loss of strawberry fruit remains a critical challenge due to its perishable nature-thin epidermis, rapid respiration, and susceptibility to mechanical injury and microbial proliferation, which accelerate senescence, energy depletion, and quality deterioration during storage and transportation, ultimately rendering them unsuitable for consumption and reducing their commercial value (3). Consequently, developing innovative preservation technologies to decelerate postharvest senescence, retain nutritional integrity, and prolong shelf life is critical for sustaining strawberry quality and reducing economic losses.

1-methylcyclopropene (1-MCP) is a potent ethylene receptor inhibitor that blocks the ethylene signaling pathway by binding to the ethylene receptor, thereby delaying the ripening and senescence of postharvest fruits and vegetables (4). Due to its cost-effectiveness, operational simplicity and economic viability, 1-MCP has been extensively applied in postharvest fruit storage (5). Accumulating studies have indicated that 1-MCP could alleviate the softening process by suppressing cell wall degradation and enhancing the antioxidant capacity in kiwifruit, apple and apricots (6–8). Moreover, 1-MCP can be synergistically integrated with other treatments, including modified atmosphere packaging and tea polyphenol coating, to improve the preservation effect of postharvest fruits and vegetables (4, 9). Yang et al. (10) found that the combined 1-MCP and chlorine dioxide treatment effectively delayed postharvest senescence and maintained the commodity quality of strawberries by increasing the ability of the antioxidant system. Furthermore, hydrogen sulfide (H_2_S), a gaseous signaling molecule akin to carbon monoxide and nitric oxide, plays a critical role in plant development, senescence, and abiotic stresses (11, 12). Recent studies have pointed out that H_2_S at low concentrations could also modulate the antioxidant system and cell wall metabolism to maintain the storage quality and extend the shelf life in postharvest fruits and vegetables, such as jujube, kiwifruit and sweet cherry (13–15). Similar to 1-MCP, Hu et al. (16) reported that H_2_S treatment enhanced antioxidant capacity might be an indispensable endogenous maturation and senescence regulating factor in strawberries. However, the regulatory mechanisms of 1-MCP and H_2_S, particularly their individual and synergistic effects on sugar metabolism and energy homeostasis, have not been fully explored in strawberry fruit.

The primary soluble sugars, such as glucose, fructose and sucrose, are intricately linked to the postharvest physiological metabolism of fruits and vegetables (17). Sucrose, glucose, and fructose not only serve as metabolic substrates for the synthesis of pigments, vitamins, and aromatic substances to enhance taste and flavor but also play vital roles in the energy supply for metabolic processes (18, 19). The homeostasis of soluble sugars is governed by enzymes including acid invertase (AI), neutral invertase (NI), sucrose synthase (SS), and sucrose phosphate synthase (SPS) (20). For instance, in apple fruit, nitroprusside (SNP) treatment enhanced sucrose accumulation by inducing the activities of SPS and SS while inhibiting the activities of AI and NI, which contributed to the quality maintenance (21). Crucially, sugar metabolism is intrinsically linked to cellular energy homeostasis, dictated by adenosine phosphates, which critically influence postharvest physiology of fruits and vegetables (18, 22). Adenosine triphosphate (ATP) levels and energy charge (EC), as pivotal biomarkers of cellular energy status, directly correlate with the activities of energy metabolism-related enzymes, including adenosine triphosphatases (ATPases), succinate dehydrogenase (SDH), and cytochrome c oxidase (CCO) (23). Increasing studies indicated that insufficient energy supply and diminished cellular energy production efficiency triggered destruction of membrane structural integrity, which may underlie physiological disorder and fruit senescence (24). Zhang et al. (25) pointed out that SO_2_ and CO_2_ co-treatment delayed the reduction of ATP and EC levels via enhancing the activities of SDH and CCO, maintaining cell energy to postpone senescence in strawberries. Similarly, exogenous nicotinamide enhanced ATP levels via mitochondrial energy metabolism, which contributed to alleviating senescence in strawberry fruit (26).

Despite these advances, the regulatory interplay of 1-MCP and H₂S, individually or synergistically, on sugar-energy metabolism in strawberry fruit remains uncharacterized. This study investigated the effects of 1-MCP and H₂S fumigation on the physiological quality, sucrose accumulation and EC levels in postharvest strawberry fruit. Meanwhile, the activities of AI, NI, SS and SPS involved in sugar metabolism and the activities of SDH, CCO and ATPases involved in energy metabolism were analyzed to elucidate the enzymatic mechanisms underlying metabolic regulation. Therefore, this work clarified how 1-MCP and H₂S treatments modulated ripening and senescence by regulating sugar and energy metabolism pathways, offering novel insights into targeted postharvest preservation strategies of strawberry fruit.

## 2 Materials and methods

### 2.1 Fruit material and treatment

Freshly harvested “Hongyan” strawberries (*Fragaria × ananassa* Duch.) at 80% commercial maturity (about 30 N firmness and 8.5% total soluble solids) were obtained from an orchard in Baohe District, Anhui Province. Fruits were transported to the laboratory within 2 h and subjected to surface sanitation using 0.1% (v/v) sodium hypochlorite solution for epiphytic microbe elimination and field heat dissipation. A total of 720 defect free fruits were selected through visual inspection and randomized into four treatments: (1) control: fumigated with distilled water; (2) 1-MCP treatment: fumigated with 1.0 μL L^−1^ of 1-MCP; (3) H_2_S treatment: exposed with 0.8 mmol L^−1^ of sodium hydrosulfide (NaHS, H_2_S donor); and (4) combination treatment: co-fumigation with 1.0 μL L^−1^ of 1-MCP and 0.8 mmol L^−1^ of NaHS. All treatments were conducted in the 85 L sealed containers for 24 h under ambient temperature. Then, every ten strawberries were kept in a plastic box (175 mm × 135 mm × 75 mm) and stored in climate controlled chambers (LISK, Nanjing, China) at 4 ± 0.5°C with a relative humidity of 80–85% for 15 d. Samples were taken at 0, 3, 6, 9, 12 and 15 d, quickly frozen with liquid nitrogen, and stored at −80°C for further biochemical analysis. Three independent biological replicates were conducted with complete treatment randomization.

### 2.2 Decay rate and decay index

The determination of decay rate and decay index was conducted following the method described by Zhang et al. (27). Decay rate (%) = (number of rotten fruits/total number of fruits) × 100%. Decay index (DI) was carried out based on the proportion of the decayed area to the entire fruit area and was divided into 4 levels: level 0 corresponded to a decayed area of 0%; level 1 corresponded to a decayed area of 1–25%; level 2 corresponded to a decayed area of 26–50%; and level 3 corresponded to a decayed area of 51–100%. The decay index was calculated as DI = ∑ [(decay level× number of fruits in that level)]/(4 × total number of fruits).

### 2.3 Weight loss rate and firmness

The determination of weight loss followed the method outlined by Zhang et al. (27). The formula for weight loss rate was weight loss (%) = [(W_0_ − W_1_)/W_0_] × 100%, where W_0_ was the initial weight (g), and W_1_ was the final weight.

Firmness was assayed following the method of Aday et al. (28) with slight modifications. Texture profile (TPA) testing was performed with a cylindrical probe with a 5 mm diameter (P/5 probe) (TA-XT plus, Stable Micro Systems, United Kingdom). Testing parameters included a pressing distance of 5 mm, pre-test speed, testing speed, and post-test probe return speed of 3 mm s^−1^, 1 mm s^−1^, and 1 mm s^−1^, respectively. The results were expressed in newton (N).

### 2.4 Sucrose, glucose and fructose contents

The contents of sucrose, glucose and fructose were determined following the method of Wang et al. (29) with 2 g frozen fruit powder. High-performance liquid chromatography (HPLC, Waters 1,525 + 2,414, United States) equipped with a hydrophilic interaction chromatography column (4.6 × 250 mm, 5 μm, Shodex Asahipak NH2P-50 4E, Japan) was applied to separate and analyze. The mobile phase was used 75% acetonitrile with a flow rate of 1 mL min^−1^. The column temperature was set at 40°C, and the injection volume was 20 μL. The results were expressed as mg g^−1^ FW (fresh weight).

### 2.5 Sucrose, glucose and fructose contents

Extraction of crude enzyme solution of soluble sugar metabolism enzymes was carried out following the method of Wang et al. (29) with slight modifications. 2 g of strawberry fruit were added to 5 mL of 0.1 mmol L^−1^ pH 7.5 phosphate-buffered saline (PBS) (containing 2.5 mmol L^−1^ dithiothreitol (DTT), 2% (w/v) polyvinylpyrrolidone (PVP), 5 mmol L^−1^ magnesium chloride (MgCl_2_), and 0.1% (v/v) Triton X-100). The mixture was centrifuged at 12000 g for 30 min at 4°C, and the supernatant was collected for later assays.

For AI activity, the reaction system included 0.4 mL of supernatant, 1.2 mL of sodium citrate buffer, and 0.4 mL of sucrose. For NI activity, the reaction system contained 0.4 mL of supernatant, 1.2 mL of PBS, and 0.4 mL of sucrose. The absorbance of the mixture was measured at 540 nm using a spectrophotometer (TU-1950, Beijing Puxitongyong Instrument Technology Co., Ltd., China). One unit of AI and NI activities were defined as the ability of an enzyme to produce 1 μmol of glucose per gram per hour. The reaction system for SS activity consisted of 80 μL of supernatant, 0.4 mL of Hepes-NaOH reaction buffer (containing 4 mmol L^−1^ uridine diphosphate glucose (UDPG), 15 mmol L^−1^ MgCl_2_ and 60 mmol L^−1^ fructose). The reaction system for SPS activity consisted of 80 μL of supernatant and 0.4 mL of Hepes-NaOH reaction buffer (containing 4 mmol L^−1^ UDPG, 15 mmol L^−1^ MgCl_2_ and 5 mmol L^−1^ fructose-6-phosphate). The absorbance was determined at 490 nm using a spectrophotometer (TU-1950, Beijing Puxitongyong Instrument Technology Co., Ltd., China). One unit of SS and SPS activities were defined as the ability of enzyme to product 1 μmol of sucrose per gram per hour. All enzymes were expressed as U g^−1^ FW.

### 2.6 ATP, ADP and AMP contents and EC

The contents of ATP, ADP, and AMP were measured according to the method of Xie et al. (30). 2 g of frozen strawberry powder were homogenized with 5 mL of ice-cold 0.6 mol L^−1^ HClO_4_ and centrifuged at 12,000 g for 20 min. 1 mol L^−1^ potassium hydroxide was used to adjust the pH of supernatant to 6.5–6.8. The mixture was then filtered through a 0.45 μm microporous membrane for HPLC analysis (Waters 2,695, United States) equipped with a UV detection. Kromasil 100-5-C18 (250 mm × 4.6 mm) column was used for chromatographic separation with a 25°C column temperature. 0.05 mol L^−1^ potassium phosphate buffer at pH 7.0 was used as the mobile phase A, while acetonitrile was set as mobile phase B, and the flow rate was 0.8 mL min^−1^. Linear gradient elution was performed with the volume percentages of mobile phase A being 100, 80 and 100% at 0, 7 and 10 min, respectively. The total elution time was 20 min, and the injection volume was 10 μL. Results were expressed as μg g^−1^ FW. EC was calculated as EC = (ATP + 0.5 ADP)/(ATP + ADP + AMP).

### 2.7 SDH, CCO, H^+^-ATPase and Ca^2+^-ATPase activities

The mitochondria were extracted following the method of Zhao et al. (31) with slight modifications. Briefly, 3 g of strawberry powder were added to 6 mL of 50 mmol L^−1^ Tris–HCl buffer (pH 7.5, containing 0.25 mol L^−1^ sucrose, 0.3 mol L^−1^ mannitol, 1 mmol L^−1^ ethylenediaminetetraacetic acid (EDTA), and 0.5% PVP). The mixture was then filtered through four layers of gauze, and the filtrate was collected at 4°C by centrifugation at 12000 g for 20 min. The pellet was washed with 10 mL of 10 mmol L^−1^ Tris–HCl buffer (pH 7.2, containing 0.25 mol L^−1^ sucrose, 0.3 mol L^−1^ mannitol, and 1 mmol L^−1^ EDTA) and centrifuged again. The pellet was suspended in 1.5 mL of 10 mmol L^−1^ Tris–HCl buffer for further enzyme activity analysis.

The activities of SDH and CCO were determined following the method described by Xie et al. (30). For SDH activity, the reaction system consisted of 2.7 mL of 0.2 mol L^−1^ potassium phosphate buffer (pH 7.4, containing 0.2 mol L^−1^ sodium succinate), 0.1 mL of 1 mmol L^−1^ 2, 6-dichloroindophenol sodium, 0.1 mL of extract and 0.1 mL of 10 mmol L^−1^ methylsulfinyl phenazine. One unit of SDH activity was defined as an increase in absorbance of 0.01 per min at 600 nm. For CCO activity, the reaction system included 0.2 mL of extract, 0.5 mL of 0.04% (w/v) cytochrome c, and 0.5 mL of 0.4% (w/v) N, N-dimethyl-p-phenylenediamine. One unit of CCO activity was defined as an increase in absorbance of 0.01 per min at 510 nm.

The activities of H^+^-ATPase and Ca^2+^-ATPase were determined following the method described by Zhao et al. (31). For H^+^-ATPase activity, the reaction system consisted of 0.3 mL of extract, 1 mL of 30 mmol L^−1^ Tris–HCl buffer (pH 8.0, containing 3 mmol L^−1^ MgSO_4_, 50 mmol L^−1^ NaNO_3_ and KCl, 0.1 mmol L^−1^ Na_3_VO_4_ and ammonium molybdate) and 0.1 mL of 30 mmol L^−1^ ATP-Tris (pH 8.0). For Ca^2+^-ATPase activity, the reaction system consisted of 0.3 mL of extract, 1 mL of 30 mmol L^−1^ Tris–HCl buffer (pH 8.0, containing 3 mmol L^−1^ Ca(NO_3_)_2_, 50 mmol L^−1^ NaNO_3_ and KCl, 0.1 mmol L^−1^ Na_3_VO_4_ and ammonium molybdate) and 0.1 mL of 30 mmol L^−1^ ATP-Tris (pH 8.0). The reaction was terminated by adding 0.2 mL of 55% trichloroacetic acid after 20 min of incubation at 37°C. One unit of H^+^-ATPase and Ca^2+^-ATPase activity was defined as the capacity to release 1 μmol of phosphate at 660 nm. All enzyme results were measured using a spectrophotometer (TU-1950, Beijing Puxitongyong Instrument Technology Co., Ltd., China) and represented as U g^−1^ FW.

### 2.8 Statistical analyses

The experiment was conducted using a completely randomized design, and all statistical analyses were performed using IBM SPSS 25 software (IBM Corporation, United States). Significant differences were analyzed through one-way analysis of variance (ANOVA) with a 95% confidence interval, and Duncan’s multiple range tests were carried out to separate means. Statistical significance was represented by *p* < 0.05. Graphs were generated using Origin 2018 software (Origin Lab Corporation, United States).

## 3 Results

### 3.1 Decay rate, DI, weight loss rate and firmness

**Figure 1:**
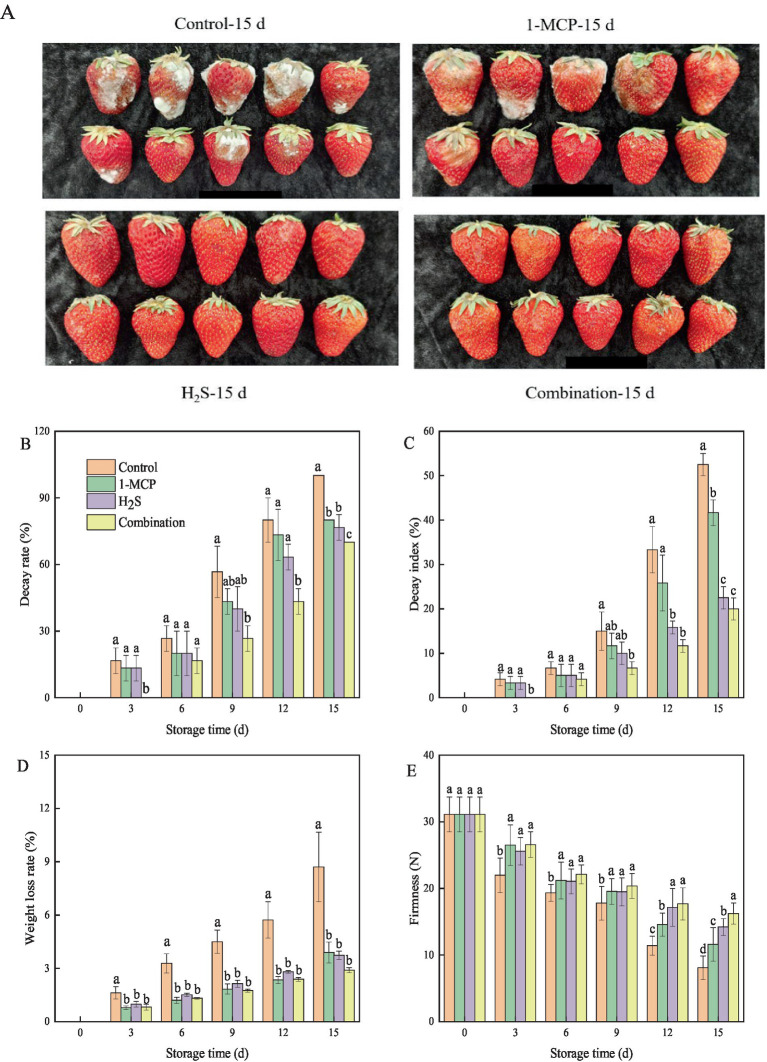
Effects of different treatments on appearance photos **(A)**, decay rate **(B)** decay index **(C)** weight loss rate **(D)** and firmness **(E)** of strawberry fruit. Data were presented as mean of triplicate samples ± standard errors. Different letters indicated significant differences between treatments.

The visual appearance and quality parameters of strawberry fruit significantly influence consumer acceptance. As demonstrated in Figure 1A, control and 1-MCP treated fruit exhibited pronounced decay and browning, whereas H₂S- and combination-treated fruit maintained relatively intact fruit surfaces. After 15 d of cold storage, both decay rate and DI of strawberry fruit increased progressively during prolonged cold storage, while all the treatments effectively attenuated these deteriorative processes compared to control (Figures 1B,C). Similarly, the weight loss rate of strawberry fruit increased with the extended cold storage time (Figure 1D). All treatments significantly (*p* < 0.05) mitigated the increase in weight loss rate in comparison with the control throughout the storage period. As shown in Figure 1E, the firmness of strawberry fruit gradually decreased during storage. Both 1-MCP and H₂S treatments effectively delayed this softening process, with H₂S exhibiting better efficacy than 1-MCP. After 15 d of storage, the firmness of strawberry fruit in the 1-MCP, H_2_S, and combination treatment was 1.4-fold, 1.7-fold, and 2.0-fold higher than that of the control, respectively.

### 3.2 Sucrose, glucose and fructose contents

**Figure 2:**
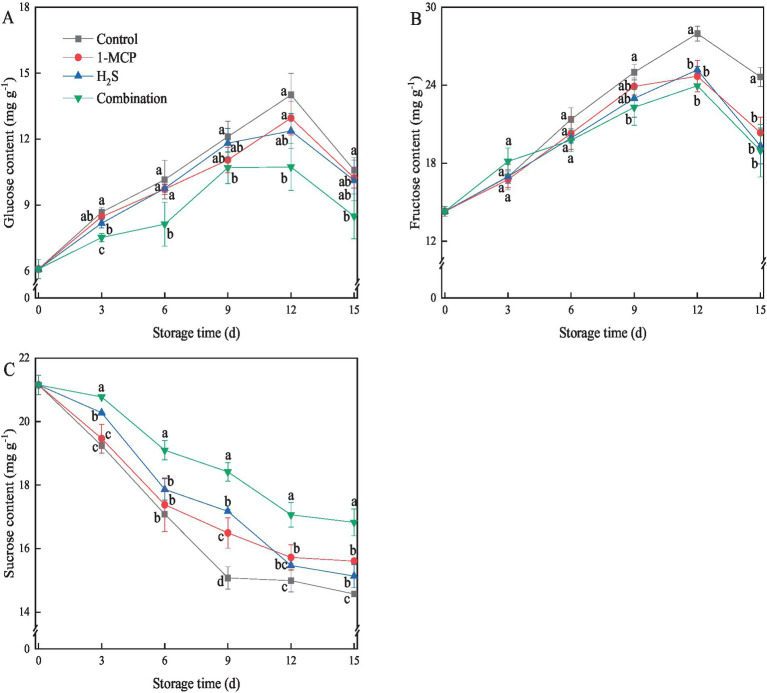
Effects of different treatments on the contents of glucose **(A)**, fructose **(B)** and sucrose **(C)** in strawberry fruit. Data were presented as mean of triplicate samples ± standard errors. Different letters indicated significant differences between treatments.

The predominant soluble sugars in “Hongyan” strawberry fruit were glucose, fructose and sucrose. As illustrated in Figures 2A,B, the level of glucose and fructose in strawberry fruit exhibited an initial increase during the first 12 d of storage, followed by a subsequent decline. All treatments effectively suppressed the increase of glucose and fructose in the later stages of storage in comparison with control, with the combination treatment maintained the lowest contents. After 15 d of storage, the content of glucose and fructose in the combination treatment was 19.8 and 23.0% lower than that of the control, respectively. In contrast, sucrose content displayed a continuous reduction throughout the whole storage time (Figure 2C), with the control showing a rapid decline before day 9 and then steadily decreased in the later stage. At the end of storage, the sucrose content in strawberry fruit treated with 1-MCP, H_2_S, and the combination treatment were 6.6, 3.7, and 13.4% higher than that of the control, respectively.

### 3.3 AI, NI, SS and SPS activities

**Figure 3:**
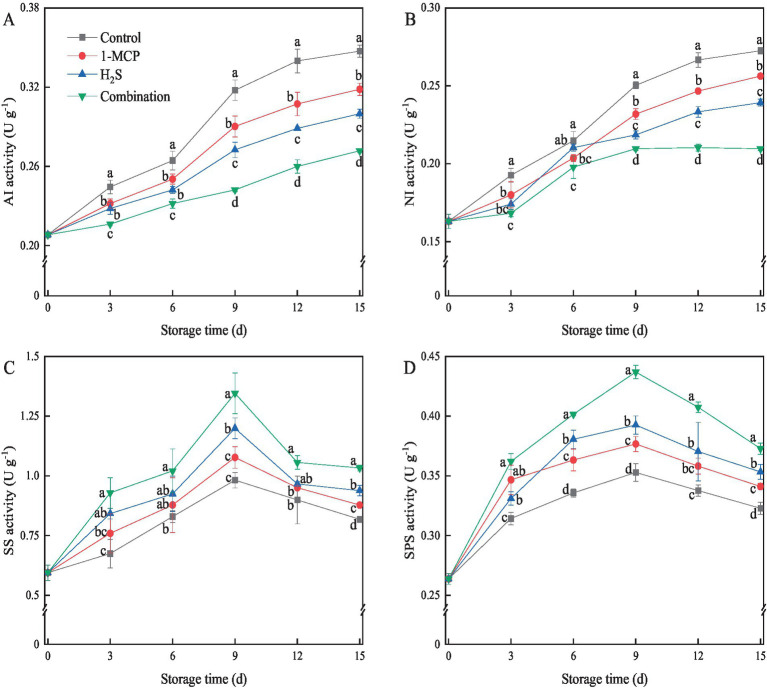
Effects of different treatments on the activities of AI **(A)**, NI **(B)**, SS **(C)** and SPS **(D)** in strawberry fruit. Data were presented as mean of triplicate samples ± standard errors. Different letters indicated significant differences between treatments.

The applied treatments modulated the activity of sugar metabolism-related enzymes in strawberry fruit during cold storage. The activities of AI and NI in strawberry fruit gradually increased with the prolongation of storage time (Figures 3A,B), and the control treatment showed the highest AI and NI activities. Meanwhile, the combination treatment inhibited the increase of AI and NI activities and maintained the lower levels in comparison with 1-MCP and H_2_S treatment after 9 d of storage. Conversely, the activities of SS and SPS in strawberry fruit initially increased and then decreased during cold storage, reaching the peak at day 9 (Figures 3C,D). The application of combination treatment significantly (*p* < 0.05) increased the activities of SS and SPS during cold storage. The activities of SS and SPS in strawberry fruit treated with 1-MCP, H_2_S and the combination treatments were 8.8 and 6.3%, 18.1 and 10.1%, 27.0 and 19.3% higher than those in control after 9 d of storage, respectively.

### 3.4 ATP, ADP and AMP contents and EC

**Figure 4:**
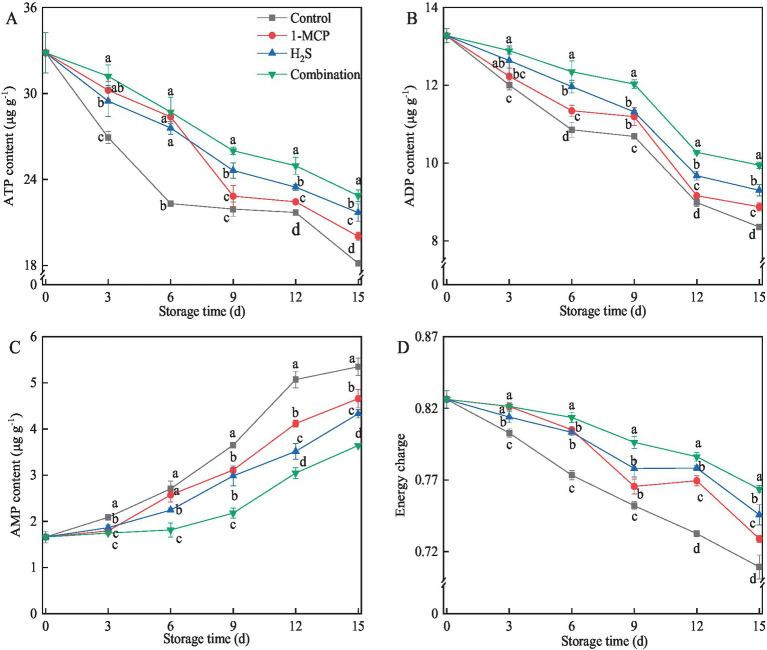
Effects of different treatments on the contents of ATP **(A)**, ADP **(B)**, AMP **(C)** and EC **(D)** in strawberry fruit. Data were presented as mean of triplicate samples ± standard errors. Different letters indicated significant differences between treatments.

Cellular energy status, a critical determinant of fruit senescence, significantly influences structural integrity during storage. As shown in Figure 4, the levels of ATP and ADP in all treatments generally exhibited progressive decline with prolonged storage time, while the level of AMP increased concomitantly. Different treatments generally alleviated and inhibited the decrease in the contents of ATP and ADP compared to the control during storage, while the combination treatment maintained the highest contents of ATP and ADP among all treatments (Figures 4B,C). On the contrary, the combination treatment suppressed the increase in AMP content, which was 32.0% lower than the control at the end of storage (Figure 4C). The influence of different treatments on the EC levels of strawberry fruit was shown in Figure 4D, which gradually decreased during the whole storage time. At the end of storage, the levels of EC in 1-MCP, H_2_S and combination treatment were 2.8%, 5.1, % and 7.7 higher than that of the control, respectively.

### 3.5 SDH, CCO, H^+^-ATPase and Ca^2+^-ATPase activities

**Figure 5:**
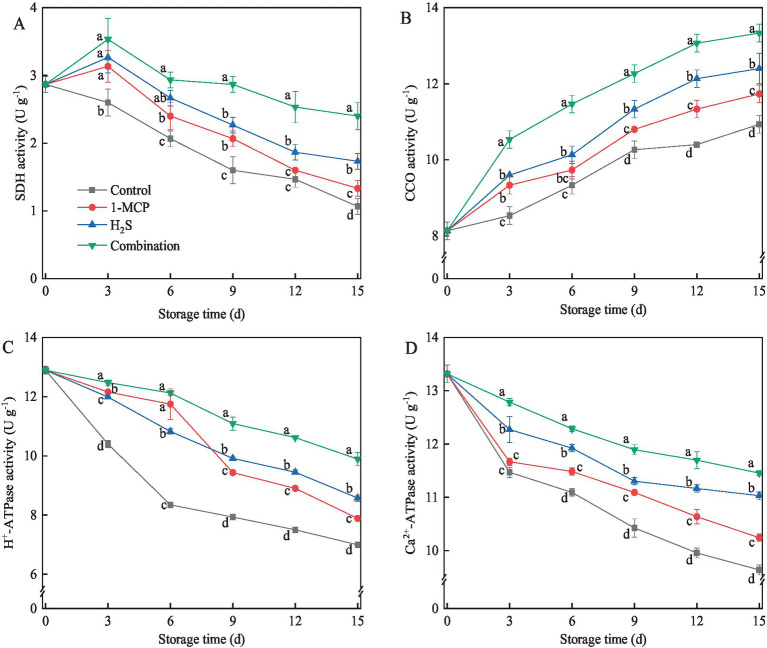
Effects of different treatments on the activities of SDH **(A)**, CCO **(B)**, H^+^-ATP **(C)** and Ca^2+^-ATP **(D)** in strawberry fruit. Data were presented as mean of triplicate samples ± standard errors. Different letters indicated significant differences between treatments.

SDH activity in treated fruit showed a trend of initially increasing and then decreasing thereafter, reaching the peak on day 3, whereas SDH activity in the control exhibited a continuous decreasing trend (Figure 5A). CCO activity in different treatments exhibited a consistent upward trend throughout the storage period (Figure 5B). Among the evaluated treatments, the combination treatment elicited the higher activities of SDH and CCO compared to H₂S treatment, while H_2_S treatment maintained higher activities than 1-MCP treatment. As shown in Figures 5C,D, the activities of H^+^-ATPase and Ca^2+^-ATPase in strawberry fruit gradually decreased during storage. The combination treatment induced higher H^+^-ATPase and Ca^2+^-ATPase activities compared to other treatments throughout the storage period. Similarly, the H₂S treatment showed higher H^+^-ATPase and Ca^2+^-ATPase activities than 1-MCP treatment after day 9. At the end of storage, the activities of SDH, CCO, H^+^-ATPase and Ca^2+^-ATPase in combination treatment were 125.0, 22.0, 41.4, and 18.9% higher than the control, respectively.

## 4 Discussion

Softening, weight loss, and decay are common changes during the ripening and senescence processes of strawberry fruits, which critically impact nutritional composition and sensory quality, and become limiting factors in shelf life and commercial value (32). Previous studies have shown that 1-MCP (an ethylene antagonist) or H_2_S (a gaseous signaling molecule) is a convenient and innovative treatment, which can effectively reduce the physiological metabolism and maintain the quality of fruits during storage (6, 12). In this study, 1-MCP or H_2_S treatment maintained the firmness while suppressing the DI and decay rate of “Hongyan” strawberry fruit, which was consistent with the results of 1-MCP-treated “Aromas” strawberry fruit (33) and H_2_S-treated “Baojiao” strawberry fruit (16). Moreover, weight loss is an important indicator of fruit quality and excessive weight loss might lead to a decrease in tissue firmness (34). The present study showed that 1-MCP or H_2_S treatment effectively inhibited the increase in weight loss, contributing to the maintenance of fruit firmness. Notably, combination treatment of 1-MCP or H_2_S in strawberries retained higher firmness and lower weight loss, DI, and decay rate compared to the 1-MCP or H_2_S treatment, indicating that the combination treatment had a synergistic effect and was more effective in maintaining fruit quality. This was consistent with previous studies on the synergistic effect of 1-MCP combined with tea polyphenols and H_2_S combined with NO to delay postharvest senescence in bracken and strawberry fruit, respectively (4, 35), thereby highlighting the potential for a 1-MCP and H₂S combined treatment to synergistically maintain strawberry fruit quality.

Soluble sugars, particularly sucrose, glucose and fructose, serve as pivotal metabolic regulators in horticultural products, orchestrating essential biochemical processes at cellular and organismal levels (19). In this study, sucrose, glucose, and fructose were identified as the primary soluble sugars in “Hongyan” strawberry fruit. Sucrose content progressively decreased while fructose and glucose levels gradually increased before day 12, mirroring the soluble sugar transition patterns observed in “Akihime” and “Tianbao” strawberry fruit (25, 36). Meanwhile, 1-MCP, H_2_S and combination treatment could effectively inhibit sucrose degradation and alleviate the increase of fructose and glucose, among which combination treatment had a more prominent effect. A prior study proposed that sucrose functioned as an antioxidant, protecting cell membranes from leakage and protein inactivation, playing an important role in the physiological metabolism of fruits (37). Elevated sucrose concentrations regulate intracellular osmotic pressure, maintaining the structure and stability of the cells (19). Therefore, different treatments might maintain high sucrose content, delay senescence induced oxidation, and maintain the cell stability of strawberry fruit. Moreover, Tokala et al. (38) found that fumigation with 1-MCP increased the sucrose content and improved the quality of apple fruit. Zhang et al. (39) observed that the application of H_2_S reduced the contents of glucose and fructose, contributing to suppressing the flavor loss of tomato fruit during storage. Consistent with these studies, the current study proved that 1-MCP or H_2_S treatment played a positive role in sucrose accumulation, while combination treatment exhibited a synergistic enhancement effect. This enhanced interaction not only stabilized membrane integrity but also modulated senescence-associated signaling pathways, thereby effectively retarding postharvest senescence processes. A similar synergistic effect was found in strawberry fruit treated with 1-MCP and ClO_2_ (10).

Sucrose metabolism is closely related to sucrose synthase and invertase enzymes (18). AI and NI act as sucrose invertase enzymes, responsible for the breakdown of sucrose to glucose and fructose, while SS and SPS act as sucrose synthesis enzymes, accounting for the biosynthesis of sucrose from glucose and fructose (29). Previous research had shown that 1-MCP treatment inhibited the accumulation of *PpAIV1* transcripts and the decrease of *PpSPS1* transcripts, thus delaying sucrose loss in Japanese pear fruit during cold storage (40). Wang et al. (29) found that peach fruit treated with glycine betaine maintained high levels of SS-synthesis and SPS activities, and low levels of AI and NI activities, resulting in the accumulation of sucrose and decrease of glucose and fructose during cold storage. In this study, 1-MCP, H_2_S and combination treatments suppressed the activities of AI and NI, while markedly enhancing the activities of SS and SPS, contributing to higher content of sucrose and lower contents of glucose and fructose in these treatments in comparison with the control. Notably, the current study found that the sucrose content in H_2_S treatment was higher than that in 1-MCP treatment before the day 9. Zhang et al. (41) demonstrated that 1-MCP directly acted as a competitive inhibitor of ethylene to inhibit the ethylene signaling pathway, which was beneficial to delaying the senescence. H_2_S, as a signaling molecule, not only antagonized the effects of ethylene by inhibiting the ethylene synthesis pathway, but also enhanced signal transduction to alleviated fruit senescence process (42). Therefore, this study speculated that H_2_S treatment in strawberry might act as signaling molecules to more actively regulate sugar metabolism than 1-MCP, affecting subsequent metabolic processes, and thus retarding the process of fruit senescence. Furthermore, the combination treatment played the most promising role in inhibiting sucrose degradation through the synergistic effect of 1-MCP and H_2_S, thereby further maintaining the quality and nutritional value of strawberry fruits during storage.

Energy metabolism is cascade-related to sugar metabolism pathways, wherein glucose and fructose serve as fundamental substrates fueling the tricarboxylic acid (TCA) cycle in the energy metabolism pathway (43). Previous evidence suggested that energy supply played an important role in maintaining membrane integrity, and insufficient cellular energy was a key factor in the ripening and senescence of fruit and vegetables (23). Huang et al. (44) found that the application of 1-MCP increased the levels of ATP content and EC, contributing to the maintenance of the cell membrane integrity and the alleviation of ripening in kiwifruit during postharvest storage. Previous studies pointed out that intracellular H_2_S was a regulatory factor for energy production to address energy demands under adverse conditions (45). In broccoli, the accumulation of ATP and EC in H_2_S treatment played vital roles in inhibiting the yellowing process and extending the postharvest shelf life (42). Similar results were found in the present study, the decrease in levels of ATP and ADP along with the increase in AMP content leading to a gradual reduction in EC in strawberry fruit. Nevertheless, 1-MCP, H_2_S, and combination treatments maintained higher ATP content to varying degrees, which helped to inhibit the reduction of EC, especially the combination treatment had the best effect. These results suggested that 1-MCP and H_2_S could mutually enhance their regulatory effects on energy metabolism. In addition, Goubern et al. (46) found that low concentrations of H_2_S could serve as electron donors to enhance ATP synthesis. These findings suggested that H_2_S might act as an electron donor to induce ATP accumulation, which accounted for the better effect than 1-MCP treatment in strawberries. Furthermore, ATP levels have been reported to participate in the regulation of antioxidant capacity and other stress responses in horticultural crops (45). Therefore, current research demonstrated that combination treatment enhanced the ATP accumulation and EC levels through synergistic effects of 1-MCP and H_2_S to regulate antioxidant capacity, which helps to maintain the cell structure integrity under the senescence process.

The activities of energy metabolism enzymes, particularly H^+^-ATPase, Ca^2+^-ATPase, SDH, and CCO, are intrinsically linked to cellular energy homeostasis and critically regulate postharvest ripening and senescence in perishable horticultural products (47). H^+^-ATPase and Ca^2+^-ATPase drive energy liberation through ATP hydrolysis into ADP and inorganic phosphate (48). Concurrently, SDH mediates the TCA cycle by catalyzing the conversion of succinate to fumarate with concomitant ATP synthesis, while CCO operates as a redox-coupled proton pump in the cytochrome c pathway to facilitate energy production (49). These inner mitochondrial membrane-bound enzymes collectively govern cellular ATP biosynthesis. Substantial evidence revealed that enzymatic suppression induced mitochondrial impairment, energy deficiency, and accelerated programmed cell death-pathological hallmarks strongly associated with accelerated fruit senescence. Liu et al. (43) established that magnetic field-mediated senescence delay correlated with sustained activation of H^+^-ATPase, Ca^2+^-ATPase, SDH, and CCO, effectively maintaining cellular EC in strawberry fruit. Complementary findings by Li et al. (42) demonstrated that enhanced enzymatic activities of those enzymes, coupled with elevated ATP levels and EC, underpin delayed senescence in broccoli. The present study showed that all treatments up-regulated these enzymatic activities while enhancing the levels of ATP and EC. The combination treatment showed a synergistic enhancement effect, which contributed to boosting mitochondrial energy production and retarding the damage of mitochondrial structure and function. This energy maintenance mechanism likely mitigated senescence-associated membrane dysfunction and structural deterioration, thereby maintaining the quality of strawberry fruit. Thus, future studies should explore the molecular mechanisms underlying this synergy, particularly its effects on ethylene signaling cross-talk, redox homeostasis, and stress-responsive pathways. The universality of combined treatment for different strawberry varieties still needs further verification.

## 5 Conclusion

In conclusion, the combined application of 1-MCP and H_2_S showed a synergistic effect in maintaining postharvest quality of strawberry fruit, which was superior to treating them separately. The combination treatment effectively inhibited the increase in DI and decay rate and alleviated the decrease of weight loss and firmness in comparison with 1-MCP or H_2_S treatment. Moreover, the combination treatment suppressed the activities of sucrose-degrading enzymes (AI and NI) while enhancing the activities of sucrose-synthesizing enzymes (SS and SPS), which contributed to the sucrose accumulation. Furthermore, higher activities of energy-metabolism related enzymes (H^+^-ATPase, Ca^2+^-ATPase, SDH, and CCO) in combination-treated strawberry fruit might be the reason for the sufficient supply of intracellular ATP and EC. These findings highlighted that the synergistic effect of 1-MCP combined with H_2_S treatment plays important roles in maintaining the quality of strawberry fruit by modulating sugar and energy metabolism pathways. Therefore, the findings of this study indicated that 1-MCP combined with H_2_S treatment could be an effective and potential method for maintaining the quality and extending the shelf life of postharvest strawberries, offering significant positive implications for advancing the strawberry industry. Furthermore, the current study provided a theoretical foundation and scientific basis for the application of 1-MCP combined with H₂S treatment in fruit and vegetable preservation.

## Data Availability

The original contributions presented in the study are included in the article/supplementary material, further inquiries can be directed to the corresponding authors.
